# The Effect of Dexmedetomidine on Postoperative Nausea and Vomiting in Patients Undergoing Thoracic Surgery-A Meta-Analysis of a Randomized Controlled Trial

**DOI:** 10.3389/fsurg.2022.863249

**Published:** 2022-03-31

**Authors:** Wei Zhang, Ruohan Wang, Bing Li, Ying Zhao, Xinmin Liu, Jingli Yuan

**Affiliations:** ^1^Department of Anesthesiology and Perioperative Medicine, Henan University People's Hospital, Henan Provincial People's Hospital, Zhengzhou, China; ^2^Department of Anesthesiology and Perioperative Medicine, Zhengzhou University People's Hospital, Henan Provincial People's Hospital, Zhengzhou, China

**Keywords:** dexmedetomidine, postoperative nausea and vomiting, thoracic surgery, meta-analysis, visual analog score

## Abstract

**Background:**

Dexmedetomidine reduces the occurrence of postoperative nausea and vomiting (PONV); however, the effect of dexmedetomidine on PONV in patients undergoing thoracic surgery remains inconclusive. In addition, the effect of different dexmedetomidine application methods, anesthetics, and surgical procedures on the effects of dexmedetomidine on PONV remains unclear. Therefore, the purpose of this meta-analysis was to study the effect of dexmedetomidine on PONV in patients undergoing thoracic surgery.

**Methods:**

Electronic databases were searched to identify randomized controlled trials studying the effects of dexmedetomidine on nausea and vomiting after thoracic surgery. In total, 12 articles that met the inclusion criteria were obtained. The primary outcome of this comprehensive analysis was the incidence of PONV; secondary outcomes included the incidence of postoperative nausea, the incidence of postoperative vomiting, postoperative visual analog score (VAS), the amount of intraoperative sufentanil, and the number of times postoperative salvage analgesia was administered.

**Results:**

Twelve trials involving 905 participants undergoing thoracic surgery were included. Compared with placebo, dexmedetomidine reduced the incidence of nausea and vomiting after thoracic surgery [12 trials; 905 participants; risk ratio (RR) = 0.32; 95% CI (0.23, 0.44); *P* < 0.00001, I^2^ = 0%]. The subgroup analysis revealed that dexmedetomidine reduces the occurrence of PONV in both thoracotomy and thoracoscopic surgery. In addition, both intravenous and local infusion of dexmedetomidine can reduce the occurrence of PONV, and intravenous or inhaled anesthetics do not affect the effect of dexmedetomidine on reducing PONV. Dexmedetomidine can reduce the postoperative resting VAS of patients, and no statistically significant differences in the amount of intraoperative sufentanil and the number of salvage analgesia procedures after surgery were noted.

**Conclusion:**

Compared with placebo, dexmedetomidine can reduce the occurrence of PONV in patients undergoing thoracic surgery, and this effect is not affected by the method of dexmedetomidine administration, use of minimally invasive surgery, and use of a combination of intravenous or inhalation anesthetics.

**Systematic Review Registration:**

https://www.crd.york.ac.uk/prospero/#myprospero, PROSPERO, identifier: CRD42021269358.

## Introduction

The risk of pulmonary complications after thoracic surgery (19–59%) is relatively high and greater than that note for upper abdominal surgery (16–17%) and lower abdominal surgery (0–5%) (1). Nausea and vomiting are the two most common adverse events after surgery. The incidence rate of postoperative nausea and vomiting (PONV) is 30% in the general population and 80% in the high-risk population (2). PONV is a very painful experience that is obviously related to patient dissatisfaction (3, 4). PONV has a serious impact on the patient's postoperative rehabilitation. It can also cause disorders based on water, electrolyte, and acid-base balance; prolong the hospital stay and increase the economic burden of the patient (5). It leads to reflux and aspiration of the patient, which subsequently increases the incidence of postoperative pulmonary complications and causes death in severe cases (1).

At present, many methods are available to prevent and treat PONV. Among them, drugs are one of the important methods. Numerous drugs can be used to reduce PONV, such as 5-HT3 receptor antagonists, neurokinin 1 (NK1) receptor antagonists, corticosteroids, and anticholinergics. These drugs have different pharmacokinetics, curative effects, and side effects (6). The incidence of PONV varies greatly among different populations. There are many studies on the prevention of PONV, but these studies are limited to specific patient populations (7, 8). Dexmedetomidine is a highly selective adrenergic receptor agonist that inhibits sympathetic nerves and provides analgesic and sedative effects with fewer side effects, such as respiratory depression (9). There are numerous ways to administer dexmedetomidine, such as intravenous, nasal, and local applications. A study by Hu et al. (10) confirmed that intravenous administration of dexmedetomidine can reduce the incidence of PONV in patients undergoing cesarean section (10). Despite statistical heterogeneity, an SRMA found reduced rates of PONV as a secondary outcome in children receiving intranasal dexmedetomidine for separation anxiety compared to intranasal or oral midazolam (11). A randomized controlled trial by Hong et al. (12) found that dexmedetomidine as an adjuvant to TPVB effectively relieves pain and significantly reduces the demand for opioids in VATS (12).

Opioids are closely related to the occurrence of PONV (2). Studies have shown that dexmedetomidine not only reduces the incidence of PONV (13, 14) but also significantly reduces postoperative pain scores and the amount of opioids (15, 16) which may reduce nausea and vomiting associated with opioids.

Different types of surgery exhibit different incidences of PONV. Laparoscopy, bariatric surgery, gynecological surgery, and cholecystectomy may be associated with an increased risk of PONV (8). In total, 25–30% of surgical patients will experience PONV, so it is a problem that anesthesiologists are concerned about (17, 18). Wang et al. (19) reported that dexmedetomidine reduces the incidence of PONV in thoracic surgery, while in a study by Cai et al. (20), dexmedetomidine exhibited no significant difference in reducing PONV compared with normal saline in thoracic surgery. A consensus on the effect of dexmedetomidine on PONV in patients undergoing thoracic surgery is lacking. Therefore, the purpose of this meta-analysis was to comprehensively explore the effect of dexmedetomidine on PONV in patients undergoing thoracic surgery.

In this meta-analysis, we comprehensively analyzed 12 randomized controlled trials assessing the effect of dexmedetomidine on PONV in patients undergoing thoracic surgery to explore the effect of dexmedetomidine on PONV in thoracic patients.

## Methods

### Study Protocol and Registration

The meta-analysis was conducted in accordance with the Cochrane Handbook for Systematic Reviews of Interventions (21) and is reported in compliance with the Preferred Reporting Items for Systematic Reviews and Meta-Analyses (PRISMA) statement (22). This study was prospectively registered in the PROSPERO registry (CRD42021269358), https://www.crd.york.ac.uk/prospero/#myprospero.

#### Inclusion Criteria

Types of trials: randomized controlled trials (RCT).
Types of participants: patients undergoing thoracic surgery.
Types of interventions: dexmedetomidine.
Types of outcome measures: the incidence of PONV, the incidence of postoperative nausea, the incidence of postoperative vomiting, postoperative visual analog score (VAS) score, the amount of intraoperative sufentanil, and the number of postoperative salvage analgesia procedures.

#### Exclusion Criteria

Animal experiments, published in non-English languages.

### Search Strategy and Quality Evaluation

#### Search Strategy

One reviewer searched for studies reported in PubMed, Embase, Web of Science, and the Cochrane Central Register of Controlled Trials through July 1, 2021, without any restrictions. Controlled vocabulary (MeSH in PubMed and Emtree in Embase) and keywords were used. Search terms included those related to PONV, dexmedetomidine, thoracic surgery, and their variants. The complete search strategy is available in additional file 1. Two reviewers hand-checked the reference lists of eligible trials and previous reviews.

#### Methodological Quality Evaluation of the Included Literature

The Cochrane Collaboration's tool (23) was used to assess the risk of bias and to evaluate the methodology of the included studies. We reviewed each trial and scored them as high, low, or unclear in terms of their risk involving the following domains: random sequence generation (selection bias), allocation concealment (selection bias), blinding of participants and personnel (performance bias), blinding of outcome assessment (detection bias), incomplete outcome data (attrition bias), selective reporting (reporting bias), and other bias. Disagreements were resolved by discussion with a third reviewer.

### Document Screening, Data Extraction, and Collection

After the records were imported into EndNote reference management software (Clarivate Analytics), duplicate records were removed. Two reviewers screened the titles and abstracts for relevance and labeled records as included, excluded, or uncertain. In the case of uncertainty, the full-text articles were retrieved to assess eligibility. Disagreements were resolved by discussion with other reviewers.

#### Data Extraction and Collection

Two reviewers independently extracted the data using a standardized form. We collected information on the trial characteristics (year of publication, number of patients), patient characteristics (age), intervention characteristics (Methods of administration of anesthetics, types of compound anesthetics), and data about the primary and secondary outcomes. Disagreements were resolved by discussion with other reviewers.

The data are expressed as the mean ± SD. If the study provided the median and interquartile range instead of the mean and SD, we calculated the mean and SD using the method developed by McGrath et al. (24).

### Data Analysis and Synthesis

#### Meta-Analysis

Review Manager (RevMan 5.3; Copenhagen: Nordic Cochrane Center Collaboration, 2014) was used for analysis. Differences are expressed as relative risks (RRs) with 95% CIs for dichotomous outcomes and standardized mean differences (SMDs) with 95% CIs for continuous outcomes. P < 0.05 indicates that the difference is statistically significant. Heterogeneity testing was performed using the Z score and X^2^ statistical analysis; P < 0.1 was considered to indicate heterogeneity. When P > 0.1 and I^2^ < 50%, the heterogeneity was ignored, and a fixed-effects model was used when I^2^ > 50%; if the heterogeneity was not easily explained, a random-effects model was selected, subgroup analysis was performed, the effect index was changed, or sensitivity analysis was conducted. Subgroup analysis was conducted based on the surgical methods, methods of dexmedetomidine administration, and types of combined anesthetics. Potential publication bias was analyzed using a “funnel plot” by the Review Manager (RevMan 5.3; Copenhagen: Nordic Cochrane Center Collaboration, 2014).

## Results

### Trial Selection and Characteristics

**Figure 1:**
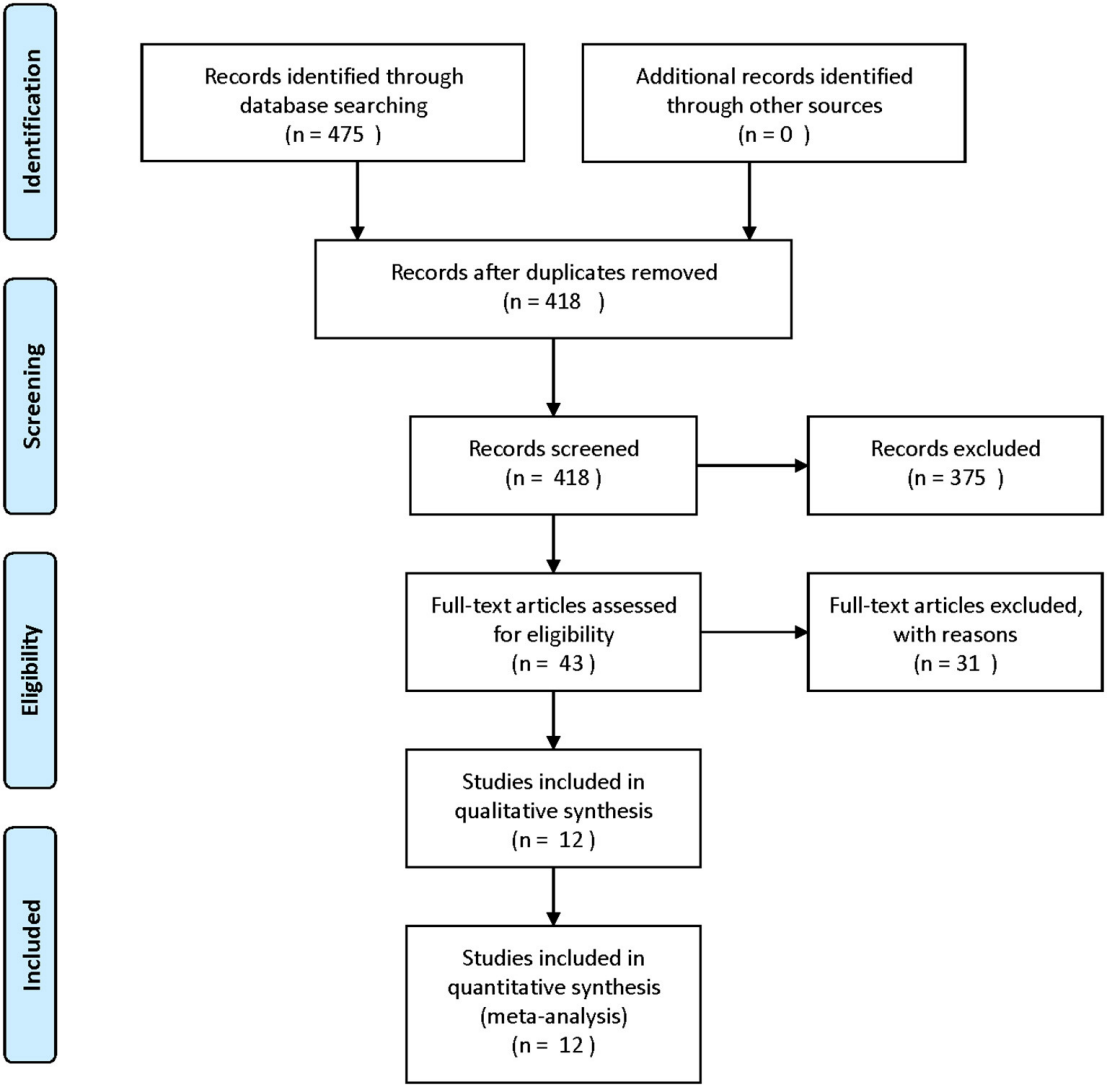
Flow diagram.

The study flow diagram is shown in Figure 1. The initial search yielded 475 records, after removing duplicates and screening the titles and abstracts, 43 articles were deemed potentially eligible. After reviewing the full-text articles, 12 trials (19, 20, 25–34) were included in the final analysis.

**Figure 2:**
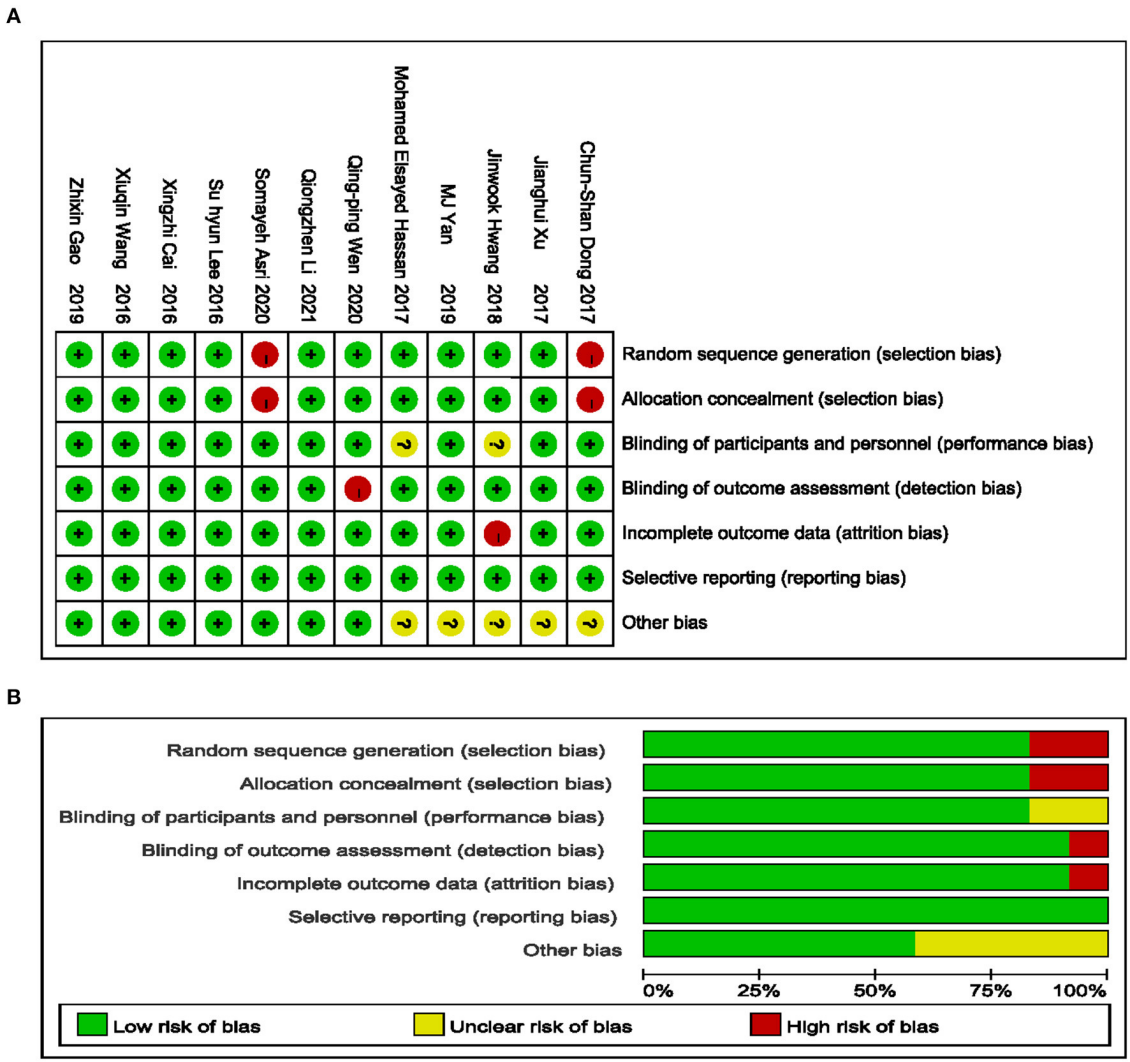
Risk of bias summary. Green low risk, yellow unclear risk, red high risk **(A)**; Green low risk of bias, yellow unclear risk of bias, red high risk of bias **(B)**.

The characteristics of the included trials are summarized in Table 1. The 12 included trials were published from 2016 to 2020 with sample sizes ranging from 41 to 143 subjects and a total of 905 subjects. Details of the risk of bias are presented in Figure 2.

**Table 1:** Characteristics of included trials.

**Study ID**	**Participants**	**Surgery types**	**Anesthesia methods**	**Comparison**	
				**Intervention group (I)**	**Control group (C)**
Hwang et al. (25)	41 patients 16–45 yr	Thoracoscopic bullectomy	C:SA GA	SA: D 1 μg/kg for 10 min maintain:D 0.3–1 μg/kg/h, ketamine 2–4 mg/kg/h	GA: maintain: S
Gao et al. (26)	90 patients 20–65 yr	VATS	GA	I1:0.5% R 30 mL with 10 mg D I2:0.5% R 30 mL with 1 μg/kg Dmaintain: P and remifentanil	C:0.5% R 30 mL maintain: P and remifentanil
Wang et al. (19)	84 patients 18–75 yr	VATLS	GA	0.5 μg/kg of D IV PCA:50 mg of oxycodone and 0.05 μg/kg/h of D diluted to 100 mL, 1 mL/h and a bolus dose of 2 mL, with a lock-out of 15 min maintain:P or S	Oxycodone 10 min IV PCA:0.5 mg/ml of oxycodone 1 mL/h and a bolus dose of 2 mL, with a lock-out of 15 minu maintain:P or S
Li et al. (27)	152 patients 30–60 yr	VATS	GA	0.5 μg/kg D 10 min PCIA: 1.5 μg/kg sufentanil and 0.3 mg/kg dezocine and 3.0 μg/kg D maintain: P	Normal saline 10 min PCIA: 1.5 μg/kg sufentanil and 0.3 mg/kg dezocine maintain: P
Yan et al. (28)	130 patients 35–60 yr	Elective open lung lobectomy	GA	PCEA: 0.5 μg/mL of D+0.1% R maintain: P and remifentanil	PCEA:0.5 μg/mL of sufentanil +0.1% R maintain: P and remifentanil
Cai et al. (20)	94 patients 18–65yr	Thoracic surgery	GA	0.25 mL/kg of 1 mg/kg D IV 10 min, 0.125 mL/kg/h (0.5 mg/kg/h D) until 30 min before the end of surgery. maintain: S.	The same volume of saline.maintain: P and remifentanil maintain: S.
Dong et al. (29)	60 patients 32–65yr	Elective major open thoracotomy	GA	PCIA: sufentanil 3.0 μg.kg−1 and 8 mg ondansetron and 4.0 μg.kg−1 of D (250 ml) maintain: P and S	PCIA: sufentanil 3.0 μg.kg−1 and 8 mg ondansetron (250 ml) maintain: P and S
Miao et al. (30)	54 patients 18–65yr	Thoracoscopic surgery	GA	D 1 μg/kg IV 10 min, 0.4 μg/kg/h D until 30 min before the end of the surgery, PCIA: 0.1 μg/kg/h D, 3 mg/kg KET, and 0.5 mg palonosetron, maintain: P	The same volume of saline, PCIA: 1.5 μg/kg SUF, 3 mg/kg KET, and 0.5 mg palonosetron, maintain: P.
Xu et al. (31)	60 patients 30–70yr	VATS	GA	75 mg/20 ml (0.375%) R + 1 μg/kg D maintain: S	75 mg/20 ml (0.375%)R maintain: S
Lee et al. (32)	100 patients 20–74yr	VATS	GA	D 1.0 μg/kg IV 20 min, maintain: desflurane	The same volume of saline, maintain: desflurane
Hassan and Mahran (33)	40 patients 18–68yr	Thoracic surgery	GA	0.25% bupivacaine + D 1 Î$\frac{1}{4}$g/kg at 0.3 mL/kg IV 5 min D 0.2 Î$\frac{1}{4}$g/kg/h + 0.125% bupivacaine 0.1 mL/kg/h. maintain: S	0.25% bupivacaineat 0.3 mL/kg IV 5 min 0.125% bupivacaine 0.1 mL/kg/h. maintain: S
Asri et al. (34)	42 patients 20–60yr	Thoracic surgery	GA	0.5 mL/kg bolusof the solution (0.3 μg/kg of D) IV 10 min, D 0.9 mL/kg/h (0.3 μg/kg/h) maintain: isoflurane	0.5 mL/kg bolus of the solution (placebo) IV 10 min, constant infusion of placebo 0.9 mL/kg/h maintain: isoflurane

*Yr, years; GA, general anesthesia; SA, sedation anesthesia; C, control; I, Intervention; D, dexmedetomidine; P, propofol; S, sevoflurane; R, ropivacaine; VATS, Video-assisted thoracic surgery; VATLS, video-assisted thoracoscopic lobectomy surgery*.

Participants in 10 trials included men and women. One study (20) exclusively enrolled women, and one study (27) exclusively focused on men. Participants underwent thoracoscopy in seven trials and thoracotomy in three trials. Dexmedetomidine was infused intravenously in 8 trials and locally in 4 trials. In 4 trials, dexmedetomidine was infused locally in combination with intravenous anesthetics. In six trials, dexmedetomidine was infused in combination with inhaled anesthetics.

### Primary Outcome

#### The Effect of Dexmedetomidine on the Incidence of PONV

##### Heterogeneity Test

The heterogeneity of the 12 trials included in this meta-analysis was assessed (I^2^ = 0%), and the value was less than the critical point of 50%. Here, the Q test *P* = 0.83 >0.1, indicating that the heterogeneity between the studies included in this meta-analysis was not statistically significant. A fixed-effects model was selected for meta-analysis.

##### Meta-Analysis Effect Value, Combined Results, and Fixed-Effect Meta-Analysis

**Figure 3:**
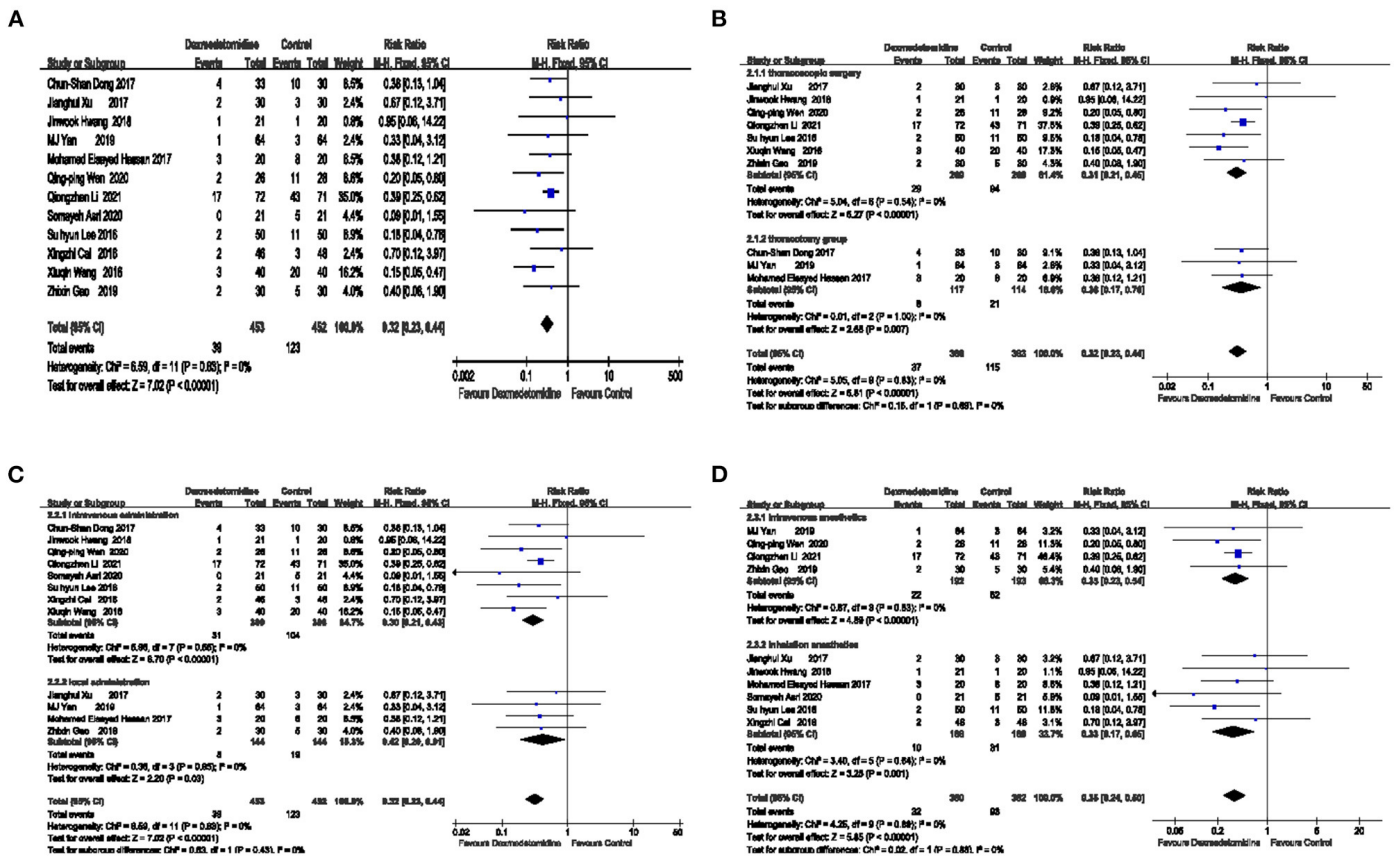
The effect of dexmedetomidine on the incidence of postoperative nausea and vomiting (PONV). The risk of PONV was lower in the dexmedetomidine group compared with the control group **(A)**. Dexmedetomidine reduced the occurrence of PONV in both thoracoscopic surgery and thoracotomy **(B)**. Dexmedetomidine reduced the occurrence of PONV regardless of whether it was intravenously or locally administered **(C)**. Dexmedetomidine reduced the occurrence of PONV regardless of whether it was combined with intravenous anesthetics or inhalation anesthetics **(D)**.

Summary results were obtained from 12 studies [RR = 0.32 95%CI (0.23, 0.44)], and the result was statistically significant (Z = 7.02 *P* < 0.00001 <0.05). These results suggest that the risk of PONV was lower in the dexmedetomidine group compared with the control group. The specific results are shown in Figure 3A.

##### Funnel Chart Analysis

Bias test: A funnel chart is used to investigate whether publication bias exists in this study. The symmetry of the funnel chart indicates no publication bias, suggesting that the publication bias of this outcome is relatively small. The specific results are shown in Supplementary Figure 1A.

##### Subgroup Analysis

The minimally invasive surgery, the types of combined anesthetics, and the method of dexmedetomidine administration were different among the 12 included articles. To explore whether these factors may affect the effect of dexmedetomidine on PONV, three subgroup analyses were performed separately (Figures 3B–D).

Regardless of whether the operation was minimally invasive, the effect of dexmedetomidine on reducing PONV was not affected, as shown in Figure 3B.

According to the different surgical methods, the patients were divided into a thoracoscopy group and a thoracotomy group. In the thoracoscopy group, the combined effect was RR = 0.31 [95% CI (0.21, 0.45)], and the effect was statistically significant (Z = 6.27, *P* < 0.00001 <0.05), suggesting that the risk of PONV in the dexmedetomidine group during thoracoscopic surgery was 31% of that in the placebo group. In the thoracotomy group, the combined effect was RR = 0.36 [95% CI (0.17, 0.76)], and the effect was statistically significant (Z = 2.68 *P* = 0.007 <0.05), suggesting that the risk of PONV in the dexmedetomidine group during thoracotomy surgery was 36% of that in the placebo group.

Based on the method of dexmedetomidine administration, the patients were divided into an intravenous administration group and a local administration group, as shown in Figure 3C.

In the intravenous administration group, the combined effect was RR = 0.30 [95% CI (0.21, 0.43)], and the effect was statistically significant (Z = 6.70, *P* < 0.00001 <0.05), suggesting that the risk of PONV in the dexmedetomidine group was 30% of that in the placebo group. In the local administration group, the combined effect was RR = 0.42 [95% CI (0.20, 0.91)], and the effect was statistically significant (Z = 2.20, *P* = 0.03 <0.05), suggesting that the risk of PONV in the dexmedetomidine group was 42% of that in the placebo group.

According to the type of compound anesthetics, the patients were divided into an intravenous anesthetics group and an inhalation anesthetics group, as shown in Figure 3D.

The combined effect of the intravenous anesthetics group was RR = 0.35 [95%CI (0.23, 0.54)], and the effect is statistically significant (Z = 4.89, *P* < 0.00001 <0.05), indicating that the risk of PONV in the dexmedetomidine group was 35% of that in the placebo group when using intravenous anesthetics. In the inhaled anesthetic group, the combined effect was RR = 0.33 [95% CI (0.17, 0.65)], and the effect was statistically significant (Z = 3.25, *P* = 0.001 <0.05), suggesting that the risk of PONV in the dexmedetomidine group was 33% of that in the placebo group.

### Secondary Results

**Figure 4:**
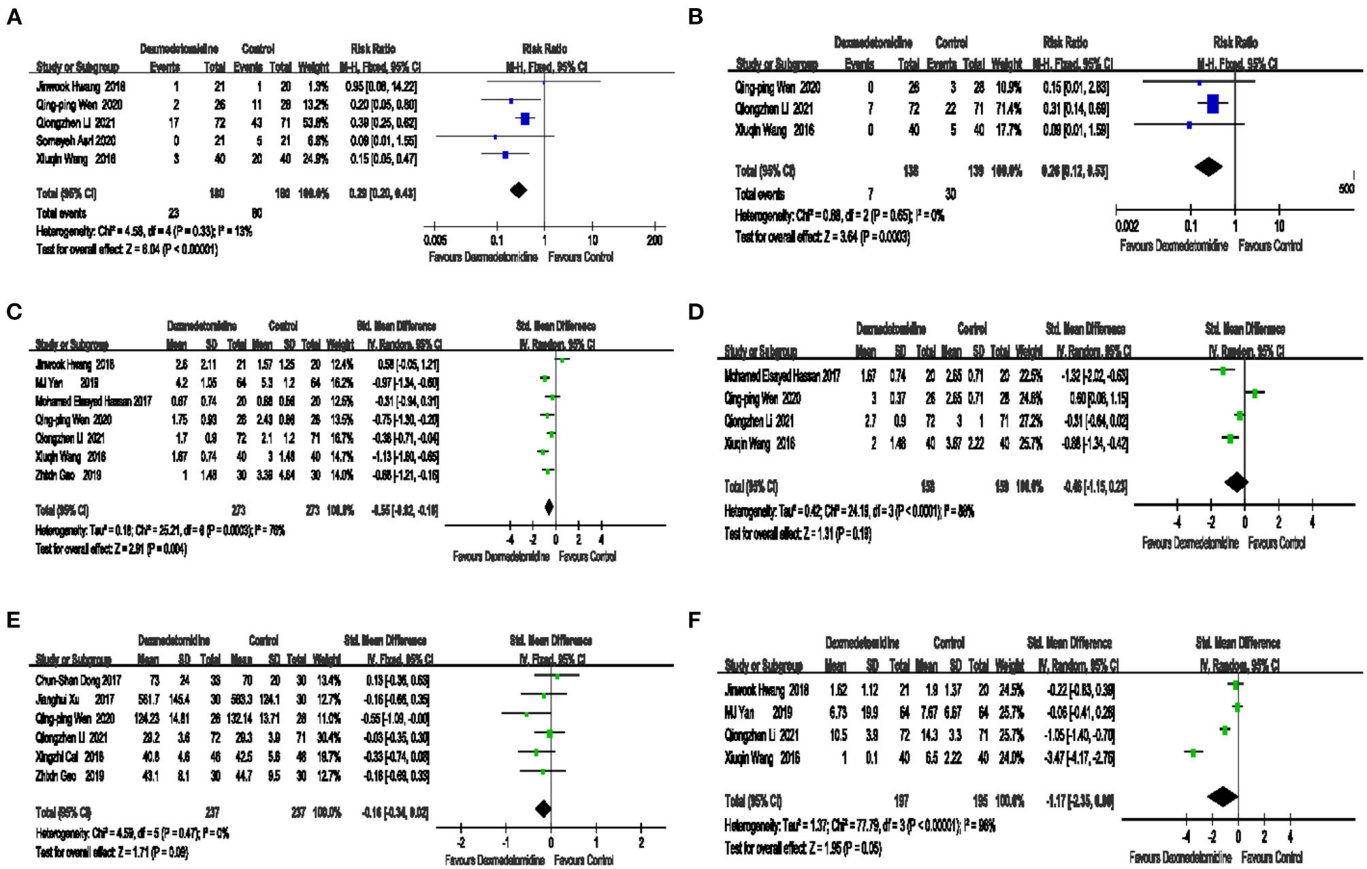
The effect of dexmedetomidine on postoperative nausea, vomiting, and pain control. Compared with placebo, dexmedetomidine significantly reduced the incidence of postoperative nausea **(A)**. The risk of postoperative vomiting in the dexmedetomidine group was significantly lower than that in the control group **(B)**. Compared with placebo, dexmedetomidine significantly reduced the postoperative visual analog score (VAS) score at rest **(C)**. No significant difference in the postoperative VAS score for coughing was noted between dexmedetomidine and placebo **(D)**. No significant difference in the amount of intraoperative sufentanil was noted between dexmedetomidine and placebo **(E)**. The number of postoperative salvage angles in procedures significantly differed between dexmedetomidine and placebo **(F)**.

The effect of dexmedetomidine on postoperative nausea is shown in Figure 4A. Compared with placebo, dexmedetomidine significantly lowered the incidence of postoperative nausea [5 trials RR = 0.29 95% CI (0.20, 0.43) *P* < 0.00001 I^2^ = 13% Figure 4A], and the publication bias of this outcome was relatively small, as shown in Supplementary Figure 1B.

The effect of dexmedetomidine on postoperative vomiting is shown in Figure 4B. The risk of postoperative vomiting, in the dexmedetomidine group was significantly lower than that in the control group [3 trials RR = 0.26, 95% CI (0.12, 0.53), *P* = 0.0003, I^2^ = 0%, Figure 4B], and the publication bias of this outcome was relatively small. These data are presented in Supplementary Figure 1C.

The effect of dexmedetomidine on the postoperative VAS score at rest is shown in Figure 4C. Compared with placebo, dexmedetomidine significantly reduced the postoperative VAS score at rest [7 trials, SMD = −0.55, 95% CI (−0.92, −0.18), *P* = 0.004, I^2^ = 76%, Figure 4C].

The effect of dexmedetomidine on the postoperative VAS score for coughing is shown in Figure 4D. No significant difference in the postoperative VAS score for coughing was noted between dexmedetomidine and placebo [4 trials, SMD = −0.46 95% CI (−1.15, 0.23), *P* = 0.19, I^2^ = 88%, Figure 4D].

The effect of dexmedetomidine on the amount of intraoperative sufentanil is shown in Figure 4E. No significant difference in the amount of intraoperative sufentanil was noted between dexmedetomidine and placebo [6 trials, SMD = −0.16, 95% CI (−0.34, 0.02), *P* = 0.09, I^2^ = 0%, Figure 4E], and the publication bias of this outcome was relatively small. These data are shown in Supplementary Figure 1D.

The effect of dexmedetomidine on postoperative salvage analgesia is shown in Figure 4F. The number of postoperative salvage analgesia procedures was administered between the dexmedetomidine and placebo groups was significantly different [6 trials, SMD = −1.17, 95% CI (−2.35, 0.00), *P* = 0.05, I^2^ = 96%, Figure 4F].

## Discussion

The objective of this meta-analysis, which included 12 articles (905 patients), was to compare the effect of dexmedetomidine on PONV in patients undergoing thoracic surgery. The results of this study show that compared with placebo, dexmedetomidine can reduce the incidence of PONV. The risk of PONV in the dexmedetomidine group was 32% of that in the placebo group.

We used Review Manager (RevMan 5.3; Copenhagen: Nordic Cochrane Center Collaboration, 2014) software for analysis, and the data are expressed as the mean ± SD. Regarding data expressed in the form of the median interquartile range, the method provided by McGrath was used to transform the data into the mean ± SD for analysis (24).

Previous studies have shown that compared with thoracotomy lobectomy, thoracoscopic lobectomy is associated with fewer in-hospital postoperative complications (35). The results of this meta-analysis show that regardless of thoracoscopic surgery or thoracotomy, dexmedetomidine can reduce the occurrence of PONV. Previous studies have shown that compared with inhaled anesthetics, intravenous anesthetics can reduce the occurrence of PONV (36). The results of this meta-analysis show that whether it is combined with inhaled anesthetics or combined with intravenous anesthetics, dexmedetomidine can reduce the occurrence of PONV. Previous studies have shown that intravenous and non-intravenous administration of dexmedetomidine offers the same duration of analgesia (37). The meta-analysis results of this study show that both intravenous administrations of dexmedetomidine and local administration can reduce PONV; thus, the effect of dexmedetomidine to inhibit PONV is not affected by its administration method.

The two groups exhibited relatively large heterogeneity in the VAS score at rest, VAS score at coughing, and the number of postoperative salvage analgesia procedures. We removed each study one by one for sensitivity analysis and found that the results did not change. In addition, the heterogeneity did not significantly change. Thus, our results were relatively stable. Heterogeneity may be due to differences in the quality of the studies included in the meta-analysis, the different analgesic programs used for patients in different studies, and patient differences in tolerance.

The development and treatment of PONV involve multiple receptor systems (38), so combination treatment with multiple drugs is more effective than that noted for a single drug. Our research results show that dexmedetomidine can reduce PONV, but there is no significant difference in the amount of intraoperative sufentanil. We hypothesize that dexmedetomidine does not completely reduce PONV by reducing the amount of sufentanil used during surgery. The potential mechanisms by which dexmedetomidine reduces PONV may include the following: (1) reducing intraoperative and postoperative pain scores, opioid consumption and inhalation anesthetic requirements, which subsequently reduce opioid-related adverse events, including PONV (39); (2) intraoperative DEX decreases noradrenergic activity as a result of binding to alpha-2 presynaptic inhibitory adrenoreceptors in the locus coeruleus, which may result in an antiemetic effect (40); and (3) the overall reduction in sympathetic outflow and catecholamine release caused by DEX. High sympathetic tone and catecholamine release may trigger PONV (17).

## Limitation

This article has the following limitations: (i) Given that the included studies did not administer a uniform dose of dexmedetomidine, the effect of different doses of dexmedetomidine on PONV in patients undergoing thoracic surgery was not explored. (ii) The effect of dexmedetomidine on PONV under a certain type of thoracic surgery could not be analyzed in detail. (iii) Data regarding whether dexmedetomidine was administered intraoperatively or postoperatively for PONV could not be obtained and analyzed.

## Conclusion

Dexmedetomidine can reduce the occurrence of PONV in patients undergoing thoracic surgery regardless of the surgical method (minimally invasive or not), the combination of anesthetic agents (intravenous or inhalation anesthesia), and method of dexmedetomidine administration (intravenous or nerve block).

## Data Availability Statement

The original contributions presented in the study are included in the article/Supplementary Material, further inquiries can be directed to the corresponding author.

## Author Contributions

WZ: designed the study. RW and BL: searched and screened relevant literature. YZ and XL: data collection. JY: completed the first draft of the manuscript. All authors have read and approved the final manuscript.

## Funding

This study was supported by grants from the Henan Province Medical Science and Technology Research Project Joint Construction Project (LHGJ20190607). The funding body played roles in the design of the study and collection, analysis, and interpretation of data and in writing the manuscript.

## Conflict of Interest

The authors declare that the research was conducted in the absence of any commercial or financial relationships that could be construed as a potential conflict of interest.

## Publisher's Note

All claims expressed in this article are solely those of the authors and do not necessarily represent those of their affiliated organizations, or those of the publisher, the editors and the reviewers. Any product that may be evaluated in this article, or claim that may be made by its manufacturer, is not guaranteed or endorsed by the publisher.

## Supplementary Material

Supplementary Figure 1 Publication bias. The publication bias of the effect of dexmedetomidine on PONV is relatively small. As noted in the figure, the left and right sides of the plot are basically symmetrical **(A–D)**.

## Abbreviations

PONV: postoperative nausea and vomiting
VAS: visual analog score
RR: relative risk
SMD: standardized mean difference
RCT: Randomized controlled trials
CI: confidence interval
NK1: neurokinin 1.
