# Supplementary material for: The Effect of Dexmedetomidine on Postoperative Nausea and Vomiting in Patients Undergoing Thoracic Surgery-A Meta-Analysis of a Randomized Controlled Trial
Source: Front Surg. 2022 Mar 31;9:863249. doi: 10.3389/fsurg.2022.863249 (PMC9008250; doi:10.3389/fsurg.2022.863249)
Supplement: Supplementary Figure 1 — Publication bias. The publication bias of the effect of dexmedetomidine on PONV is relatively small. As noted in the figure, the left and right sides of the plot are basically symmetrical (A–D). [file Data_Sheet_1.ZIP › supplementary material presentation/additional file 1.docx]

Pubmed

Search: ****((("Thoracic Surgery"[Mesh]) OR (((((Surgery, Thoracic[Title/Abstract]) OR (Surgery, Cardiac[Title/Abstract])) OR (Surgery, Heart[Title/Abstract])) OR (Heart Surgery[Title/Abstract])) OR (Cardiac Surgery[Title/Abstract]))) AND (("Thoracic Surgery"[Mesh]) OR (((((Surgery, Thoracic[Title/Abstract]) OR (Surgery, Cardiac[Title/Abstract])) OR (Surgery, Heart[Title/Abstract])) OR (Heart Surgery[Title/Abstract])) OR (Cardiac Surgery[Title/Abstract])))) AND (("Postoperative Nausea and Vomiting"[Mesh]) OR ((((((((((PONV[Title/Abstract]) OR (Nausea[Title/Abstract] AND Vomiting, Postoperative[Title/Abstract])) OR (Vomiting, Postoperative[Title/Abstract])) OR (Postoperative Emesis[Title/Abstract])) OR (Postoperative Vomiting[Title/Abstract])) OR (Emesis, Postoperative[Title/Abstract])) OR (Emeses, Postoperative[Title/Abstract])) OR (Postoperative Emeses[Title/Abstract])) OR (Postoperative Nausea[Title/Abstract])) OR (Nausea, Postoperative[Title/Abstract])))****

****Embase****

- #1 thoracic AND ('surgery'/exp OR surgery)
- #2 'surgery, thoracic':ab,ti OR 'surgery, cardiac':ab,ti OR 'surgery, heart':ab,ti OR 'heart surgery':ab,ti OR 'cardiac surgery':ab,ti
- #3 #1 OR #2
- #4 Dexmedetomidine
- #5 'mpv-1440':ab,ti OR 'mpv 1440':ab,ti OR 'mpv1440':ab,ti OR 'precedex':ab,ti OR 'dexmedetomidine hydrochloride':ab,ti OR 'hydrochloride, dexmedetomidine':ab,ti
- #6 #4 OR #5
- #7 postoperative AND nausea AND vomiting
- #8 'ponv':ab,ti OR 'nausea and vomiting, postoperative':ab,ti OR 'vomiting, postoperative':ab,ti OR 'postoperative emesis':ab,ti OR 'postoperative vomiting':ab,ti OR 'emesis, postoperative':ab,ti OR 'emeses, postoperative':ab,ti OR 'postoperative emeses':ab,ti OR 'postoperative nausea':ab,ti OR 'nausea, postoperative':ab,ti
- #9 #7 OR #8
- #10 #3 AND #6 AND #9

****Cochrane****

- #1 Thoracic Surgery
- #2 (Surgery, Thoracic):ti,ab,kw OR (Surgery, Cardiac):ti,ab,kw OR (Surgery, Heart):ti,ab,kw (Heart Surgery):ti,ab,kw or (Cardiac Surgery):ti,ab,kw
- #3 Dexmedetomidine
- #4 (MPV-1440):ti,ab,kw OR (MPV 1440):ti,ab,kw OR (MPV1440):ti,ab,kw OR (Precedex):ti,ab,kw OR (Dexmedetomidine Hydrochloride):ti,ab,kw OR (Hydrochloride, Dexmedetomidine):ti,ab,kw
- #5 #1 OR #2
- #6 #3 OR #4
- #7 Postoperative nausea and vomiting
- #8 (PONV):ti,ab,kw OR (Nausea and Vomiting, Postoperative):ti,ab,kw OR (Vomiting, Postoperative):ti,ab,kw OR (Postoperative Emesis):ti,ab,kw OR (Postoperative Vomiting):ti,ab,kw OR (Emesis, Postoperative):ti,ab,kw OR (Emeses, Postoperative):ti,ab,kw OR (Postoperative Emeses):ti,ab,kw OR (Postoperative Nausea):ti,ab,kw OR (Nausea, Postoperative):ti,ab,kw
- #9 #7 OR #8
- #10 #5 AND #6 AND #9

Wos

- #1 TS=(Thoracic Surgery OR Surgery, Thoracic OR Surgery, Cardiac OR Surgery, Heart OR Heart Surgery OR Cardiac Surgery)
- #2 TS=(Dexmedetomidine OR MPV-1440 OR MPV 1440 OR MPV1440 OR Precedex OR Dexmedetomidine Hydrochloride OR Hydrochloride, Dexmedetomidine)
- #3 TS=(Postoperative nausea and vomiting OR PONV OR Nausea and Vomiting, Postoperative OR Vomiting, Postoperative OR Postoperative Emesis OR Postoperative Vomiting OR Emesis, Postoperative OR Emeses, Postoperative OR Postoperative Emeses OR Postoperative Nausea OR Nausea, Postoperative)
- #4 #3 AND #2 AND #1
