# Supplementary material for: The Effect of Dexmedetomidine on Postoperative Nausea and Vomiting in Patients Undergoing Thoracic Surgery-A Meta-Analysis of a Randomized Controlled Trial
Source: Front Surg. 2022 Mar 31;9:863249. doi: 10.3389/fsurg.2022.863249 (PMC9008250; doi:10.3389/fsurg.2022.863249)
Supplement: Supplementary Figure 1 — Publication bias. The publication bias of the effect of dexmedetomidine on PONV is relatively small. As noted in the figure, the left and right sides of the plot are basically symmetrical (A–D). [file Data_Sheet_1.ZIP › supplementary material presentation/the brief summary.docx]

The brief summary

The incidence rate of PONV is 30% in the general population and 80% in the high-risk population.There are many studies on the prevention of PONV, but these studies are limited to specific patient populations. In a word, Dexmedetomidine reduces the occurrence of postoperative nausea and vomiting (PONV); however, the effect of dexmedetomidine on PONV in patients undergoing thoracic surgery remains inconclusive. In addition, the effect of different dexmedetomidine application methods, anesthetics and surgical procedures on the effects of dexmedetomidine on PONV remain unclear. Therefore, the purpose of this meta-analysis was to study the effect of dexmedetomidine on PONV in patients undergoing thoracic surgery.

In the meta-analysis we included twelve articles (905 patients), and compared the effect of dexmedetomidine on PONV in patients undergoing thoracic surgery. Compared with placebo, dexmedetomidine reduced the incidence of nausea and vomiting after thoracic surgery (12 trials; 905 participants; risk ratio (RR) = 0.32; 95% [confidence](C:/Program%20Files%20(x86)/Youdao/Dict/8.9.9.0/resultui/html/index.html#/javascript:;) [interval](C:/Program%20Files%20(x86)/Youdao/Dict/8.9.9.0/resultui/html/index.html#/javascript:;) (CI) [0.23, 0.44]; P < 0.00001, I^2^ = 0%)

In the meta-analysis we concluded that compared with placebo, dexmedetomidine can reduce the occurrence of PONV in patients undergoing thoracic surgery, and this effect is not affected by the method of dexmedetomidine administration, use of minimally invasive surgery, and use of a combination of intravenous or inhalation anesthetics.

This finding may help reduce the incidence of postoperative nausea and vomiting, improve the postoperative satisfaction of patients, and speed up postoperative recovery.
