# Supplementary figures and images for: The Effect of Dexmedetomidine on Postoperative Nausea and Vomiting in Patients Undergoing Thoracic Surgery-A Meta-Analysis of a Randomized Controlled Trial
Source: Front Surg. 2022 Mar 31;9:863249. doi: 10.3389/fsurg.2022.863249 (PMC9008250; doi:10.3389/fsurg.2022.863249)

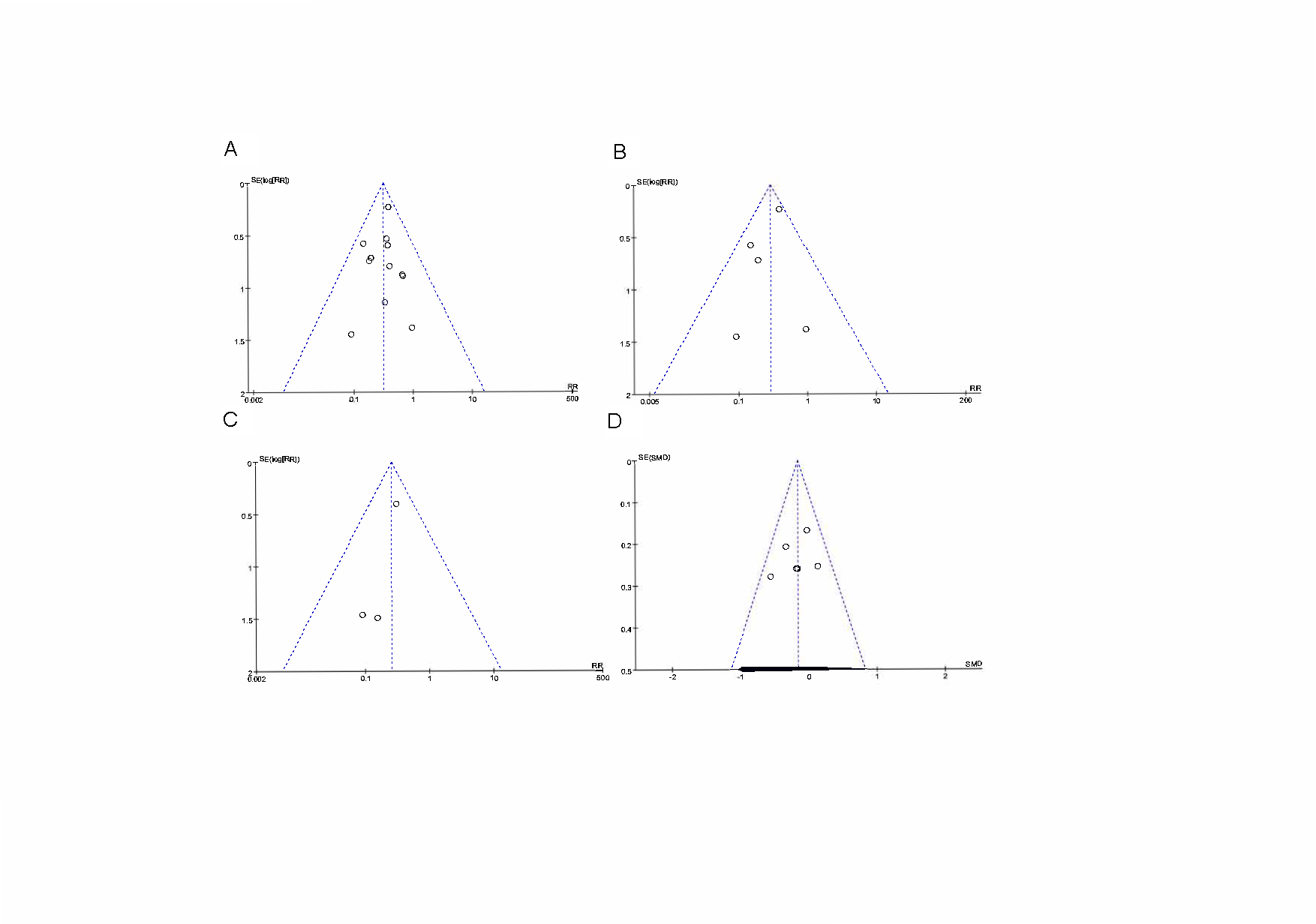

Supplement: Supplementary Figure 1 — Publication bias. The publication bias of the effect of dexmedetomidine on PONV is relatively small. As noted in the figure, the left and right sides of the plot are basically symmetrical (A–D). [file Data_Sheet_1.ZIP › supplementary material presentation/SFig 1.tif]
