# Supplementary material for: The Effect of Dexmedetomidine on Postoperative Nausea and Vomiting in Patients Undergoing Thoracic Surgery-A Meta-Analysis of a Randomized Controlled Trial
Source: Front Surg. 2022 Mar 31;9:863249. doi: 10.3389/fsurg.2022.863249 (PMC9008250; doi:10.3389/fsurg.2022.863249)
Supplement: Supplementary Figure 1 — Publication bias. The publication bias of the effect of dexmedetomidine on PONV is relatively small. As noted in the figure, the left and right sides of the plot are basically symmetrical (A–D). [file Data_Sheet_1.ZIP › supplementary material presentation/cover letter.doc]

**Dear Editors and Reviewers**:

Here with I am submitting a manuscript of research article entitled “ The effect of dexmedetomidine on postoperative nausea and vomiting in patients undergoing thoracic surgery-A meta-analysis of a randomized controlled trial” to you for consideration for publishing in “frontiers in surgery”. I would like to declare on behalf of my co-authors that the work described is original research that has not been published previously, and has no conflict of interest in the submission of this manuscript. All the authors agree with and are responsible for the data presented. All the authors listed have approved the manuscript that is enclosed. We declare that there are not any potential conflicts of interest including commercial relationships such as consultation and equity interests.

The article comprehensively [analyse](../../../../C:/Program%20Files%20(x86)/Youdao/Dict/8.9.9.0/resultui/html/index.html#/javascript:;)d the effects of dexmedetomidine on postoperative nausea and vomiting in patients undergoing thoracic surgery,  we ensure the integrity of the data and the results are authoritative, so it is worth to be published in World journal of surgical oncology. We deeply appreciate your consideration of our manuscript, and we look forward to receiving comments from you and reviewers. If you have any queries, please don’t hesitate to contact me at the address below.

Sincerely,

Jingli Yuan.

Department of Anesthesia and perioperative medicine, Henan University People’s Hospital、Henan Provincial People’s Hospital

Zhengzhou 450003

P.R China

E-mail: 2756365988@qq.com

Tel: +86-18339908398
